# Dynamics of the Dissipation of Acetamiprid, Azoxystrobin, and β-Cyfluthrin in Jalapeño Pepper (*Capsicum annuum* L.) Produced Under Greenhouse and Open-Field Conditions

**DOI:** 10.3390/foods14061023

**Published:** 2025-03-17

**Authors:** Luis Alfonso Jiménez-Ortega, Jaime Villa-Bojórquez, Pedro de Jesús Bastidas-Bastidas, Rosalba Contreras-Martínez, José Armando Carrillo-Fasio, Manuel Alonzo Báez-Sañudo

**Affiliations:** Centro de Investigación en Alimentación y Desarrollo, AC. Carretera a Eldorado Km 5.5, Campo El Diez, Culiacán 80110, Sinaloa, Mexico; ljimenez222@estudiantes.ciad.mx (L.A.J.-O.); jaimevilla89@hotmail.com (J.V.-B.); rcontreras@ciad.mx (R.C.-M.); acarrillo@ciad.mx (J.A.C.-F.)

**Keywords:** pesticides, dissipation kinetics, *Capsicum annuum*, residues, UHPLC

## Abstract

Pepper is one of the most widely consumed foods around the world. China is the leading producer, while Mexico is the primary exporter. To support these roles, the responsible use of agrochemicals is essential. Additionally, investigating the factors influencing pesticide dissipation is critical to ensure that residue levels do not exceed established Maximum Residue Limits (MRLs) and to achieve the required pre-harvest interval (PHI). This is essential to prevent trade-related issues and mitigate potential health risks to consumers. Consequently, this study aims to evaluate the dissipation dynamics of acetamiprid, azoxystrobin, and β-cyfluthrin residues in jalapeño peppers cultivated under both greenhouse and open-field conditions. Three applications of a manufacturer’s suggested dosage were evaluated, with 7-day intervals between each. The residual content was quantified after 1 h and 1, 3, 7, 14, and 21 days following each application. A QuEChERS method utilizing ultra-high-performance liquid chromatography coupled with tandem mass spectrometry (UHPLC-MS/MS) and gas chromatography equipped with a micro electron capture detector (GC-µECD) to determine the pesticide residues was optimized and validated, obtaining suitable performance, with satisfactory linearity, detection and quantification limits, recovery rates, and accuracy. The dissipation curves were constructed from the residues and dissipation percentages of the pesticides over time, elucidating the initial residuality, accumulation, half-life, residence time, and total persistence of the active ingredient. In addition, an analysis was carried out, relating climatic conditions to the cumulative dissipation of pesticides. The results show an increase in the initial residues, half-life, and residence time of pesticides in the greenhouse. Otherwise, in the open field, the residues of the pesticides acetamiprid and azoxystrobin increased over the initial applications. Climatic conditions, mainly evapotranspiration during crop growing, involve the dissipation of pesticides in jalapeño pepper. The validation method demonstrated satisfactory parameters, aligning with the guidelines provided by the US EPA and SENASICA. All concentrations quantified in real samples were found to be below the MRLs, ensuring compliance with regulatory standards. Additionally, the dissipation kinetics played a critical role in elucidating key aspects such as residence times, latency periods, and marketing timelines for ensuring food safety. This kinetics provided essential insights into the behavior and persistence of the residues, contributing to a more comprehensive understanding of their dynamics in agricultural and commercial contexts. We believe these findings underscore the reliability and applicability of the method for monitoring pesticide residues in real-world scenarios.

## 1. Introduction

Under good agricultural practices (GAPs), pesticide use is a justified act in the management of pests in crops. Pesticides and other agrochemicals are essential for managing pests and ensuring successful production. They help to prevent and eliminate pests, enhance soil fertility, improve fruit yield and quality, and, ultimately, lead to more excellent and better production. However, their excessive and unregulated use can cause harm to the environment as well as the health of users and consumers [1,2].

Consequently, the maximum residue limits (MRLs) have been set to ensure that food contains concentrations that do not pose a risk to human health. These limits are established based on good agricultural practices (GAPs), which specify the maximum allowable doses, frequencies, and intervals for pesticide application. The MRLs aim to minimize residues in food products intended for human and animal consumption [3]. To establish the MRLs, it is essential to consider the type of production technology (greenhouse or open field) because the pesticide dissipation kinetics is influenced by various environmental, biochemical, and physicochemical factors such as volatilization, hydrolysis, leaching, humidity, temperature, solar radiation, solubilization, evapotranspiration, and other chemical reactions. These processes depend directly on the environment and climatic conditions of the production site [4,5].

**Figure 1:**
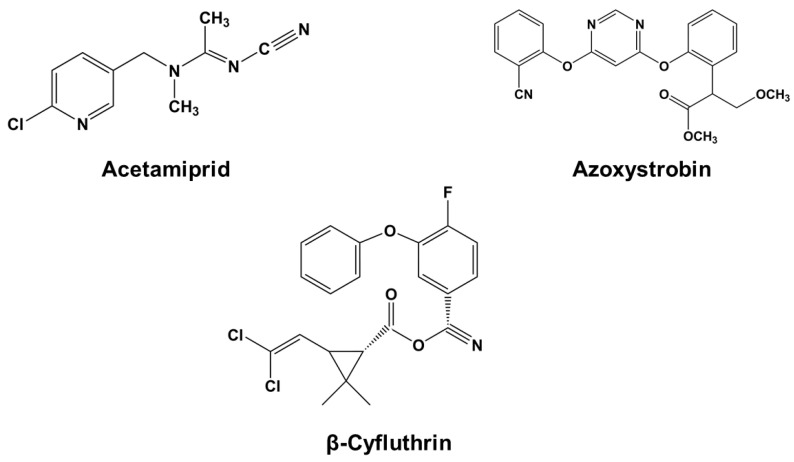
Molecular structures of model analytes studied in the present investigation.

Pepper is a fundamental and nutritional food in Asian and Latin cuisine. Moreover, it is widely appreciated and in great demand in international markets. Mexico is in first place in exports and fourth in production, only surpassed by China, Turkey, and Indonesia [6]. In 2022, Mexico produced 3,112,480.69 tons, valued at $ 36,585,968.35; however, by 2030, production is estimated to increase to more than 4.49 million tons, of which 2.11 million is estimated to be exported, representing a commercial value of 4108.98 million dollars [7,8]. The jalapeño pepper (spicy) is the most produced variety, with 703,420 tons. Sinaloa state is the second-largest producer, standing out for its quality and yield (42.08 tons/ha) [8]. Some of the pesticides, mainly used for pest control in jalapeño pepper, are acetamiprid, which is a broad-spectrum neonicotinoid insecticide, β-cyfluthrin, a pyrethroid insecticide, and azoxystrobin, a broad-spectrum systemic strobilurin-type fungicide (Figure 1).

The chemical nature of the analytes allows for the use of various analytical methodologies for their identification and quantification. For example, acetamiprid, with its hydrophilic properties, is highly soluble in water and polar solvents such as acetone and methanol. This makes its determination more convenient and efficient using liquid chromatography (LC) systems. Additionally, due to its thermal instability, analysis using gas chromatography (GC) is not recommended. Extensive research supports the use of LC as the most effective and reliable method for analyzing acetamiprid [9,10].

On the other hand, azoxystrobin, characterized by its medium polarity, is soluble in acetone, methanol, and ethyl acetate. Due to its low volatility and tendency to decompose at high temperatures, LC remains the most suitable and reliable analytical option for its determination [11,12,13]. In contrast, β-cyfluthrin is a highly hydrophobic synthetic pyrethroid that exhibits limited solubility in water and only partial solubility in organic solvents [14]. Due to its high volatility and poor ionizability, LC is not a suitable analytical technique for β-cyfluthrin analysis. In contrast, GC is widely recognized and extensively used for the determination of β-cyfluthrin and other pyrethroids [15,16,17]. GC is particularly effective because it can separate β-cyfluthrin from other pyrethroids and complex mixtures. It is ideally suited for non-polar matrices and analytes, offering high sensitivity and selectivity. Moreover, the European Food Safety Authority (EFSA) [18] recommends the use of GC for the determination of β-cyfluthrin in various food, soil, and water matrices. On the other hand, Oliveira et al. [19] mention that GC is commonly employed for the analysis of pyrethroids due to their volatile nature, which allows for their direct determination without the need for a derivatization step. Additionally, electron-capture detection (ECD) is widely used for pyrethroid detection, as it is highly sensitive to the halogen and nitrile groups present in their molecular structure. Furthermore, Tian et al. [20] mention that GC is increasingly gaining popularity in routine pyrethroid residue analysis due to its reduced susceptibility to co-matrix effects, enabling more sensitive and accurate identification. Additionally, it offers cost advantages in terms of reagents compared to LC.

Although the GAP, MRL, and ADI (admissible daily intake) (Table 1) of these pesticides have been established, it is necessary to investigate their dissipation kinetics under actual production conditions in Mexico, both in the greenhouse and in the open field, and inquire into how climatic conditions interact in its dissipation, half-life, and permanence. To guarantee that pesticide residues are below the tolerance thresholds and the edible parts have become safe for human consumption, it is important to monitor them on vegetable and fruit plants after application to corroborate the waiting period between application and harvest (pre-harvest interval (PHI)).

The dissipation of pesticides in pepper has previously been investigated [5]; however, to the best of our knowledge, this is the first study on the dissipation of pesticides in jalapeño pepper. Noteworthy, the jalapeño is one of the most exported and widely marketed pepper varieties globally. Therefore, it is essential to study the degradation of pesticides under actual production conditions in Mexico, the main exporter country worldwide. This study also takes into account the local climatic conditions and the various types of production methods, i.e., open-field and greenhouse cultivation, to obtain results that will help establish safe latency periods and maximum application doses, ensuring food safety. Therefore, the present investigation aimed to determine the kinetics dynamics of the dissipation of acetamiprid, azoxystrobin, and β-cyfluthrin in jalapeño pepper produced in a greenhouse and in open fields, in Sinaloa, Mexico.

## 2. Materials and Methods

### 2.1. Pesticide Standards and Reagents

Analytical standards for acetamiprid (C_10_H_11_ClN_4_), azoxystrobin (C_22_H_17_N_3_O_5_), and β-cyfluthrin (C_22_H_18_C_l2_FNO_3_) (>98%) were obtained from Chem Service, Inc. (West Chester, PA, USA). Water, methanol, acetonitrile-grade HPLC/MS, anhydrous magnesium sulfate, sodium chloride, sodium acetate, formic acid, and ammonium formate, ACS grade, were obtained from J.T Baker (Radnor Township, PA, USA). The primary and secondary amines (PSAs) were obtained from Agilent Technologies (Santa Clara, CA, USA).

### 2.2. Crop Development and Agronomic Management

The hybrid seeds of jalapeño pepper variety 5010 (Seminis, St. Louis, MO, USA) were sown in polystyrene trays. Peat moss was used as a germination substrate. After 40 days of growth, one batch of seedlings was transplanted into a 720 m^2^ plastic greenhouse, and another batch was transplanted into an open field under standard commercial production conditions. The substrate used in the greenhouse was coconut fiber placed in cylindrical plastic bags of 1 m length with a volume of 30 L. Two rows of bags were arranged per 10 m bed with three plants in each bag. In open fields, they were transplanted directly into clay soil in simple rows with 30 cm of separation between plants and 1.8 m between furrows. During the development of the greenhouse crop, plant management tasks were carried out using steel stakes and wooden stakes in the open field. In both production systems, a polyethylene thread was used to keep the crop upright during its growth; in the same way, irrigation was carried out by drip, jointly applying fertilization based on Steiner solution [21], with an electrical conductivity of 2.0 dS/cm and a pH of 5.5.

### 2.3. Pesticide Application and Sampling

A mixture of the pesticides acetamiprid, azoxystrobin, and β-cyfluthrin was applied at the concentrations suggested by the manufacturer (Table 1). They were applied using a motorized sprinkler (SHP-800 ECHO, Oakwood Rd., Lake Zurich, IL, USA) with two outlets. Three applications were made during the productive stage at intervals of seven days between applications. The sampling was conducted one hour after the applications and after 1, 3, 5, 7, and 14 days. In each sampling, 3 kg of pepper fruit was randomly collected, transported to the laboratory in polyethylene bags, and kept at −20 °C until use.

### 2.4. Measurement of Climatic Conditions During Pesticide Application

In the greenhouse, relative humidity, temperature, light radiation, and evapotranspiration (ET_0_) were measured every 15 min on the days of pesticide application using a HOBO data logger (ONSET, Bourne, MA, USA); and the data were analyzed in the software BoxCar Data Logger v.4.3 (ONSET, Bourne, MA, USA). In the open field, the data were obtained at the same conditions described previously using a Campbell Scientific climate station.

### 2.5. Sample Preparation and Application of the QuEChERS Method

The fruits were thawed at room temperature and homogenized in a 17 L industrial blender. A total of 15 g was recovered for pesticide analysis. The extraction and purification were performed using a QuEChERS (quick, easy, cheap, effective, rugged, and safe) method proposed by Lehotay et al. (2007) [22]. To extract the pesticides, 15 ± 0.05 g of each sample was weighed and placed in a 50 mL centrifuge tube. Next, 15 mL of acetonitrile acidified with 1% acetic acid was added and sonicated in an ultrasonic bath for 10 min. Then, 1.5 g of anhydrous sodium acetate and 6 g of anhydrous magnesium sulfate were added, mixed with a vortex at maximum speed for 1 min, and centrifuged at 5000 rpm for 5 min. For purification, 8 mL of the supernatant obtained from the previous process was taken and placed in a centrifuge tube, and 400 mg of PSA and 1.2 g of anhydrous magnesium sulfate were added. The mixture was vortexed for 1 min and centrifuged at 5000 rpm for 5 min. For LC analysis, 100 µL of the purified supernatant was taken and diluted in 900 µL of the mobile phase (mobile phase A: water acidified with 5 mM ammonium formate, pH 3.0. Mobile phase B: methanol acidified with 5 mM ammonium formate and 0.1% formic acid). For GC analysis, 4 mL of the purified supernatant was taken and concentrated on a rotary evaporator until dry and resuspended with 1 mL of acetone.

### 2.6. Identification and Quantification of Acetamiprid, Azoxystrobin Using UHPLC-MS/MS, and β-Cyfluthrin Using GC-uECD

#### 2.6.1. UHPLC-MS/MS

Waters Acquity class H-type UHPLC chromatography was used, coupled to a Waters Xevo TQ-S tandem mass spectrometer. The sample was injected (5 µL) using a Sample Manager FTN Acquity autosampler. An Acquity BEH C18 1.7 µm, 2.1 × 50 mm column was used. The mobile phase used was described in the previous section, which was used with the following gradient: initial time—5.49 min: 90% A and 10% B; 5.50–7.50 min: 10% A and 90% B; 7.51–9.0 min: 90% A and 10% B. The ionization mode used was electrospray in positive mode (ESI+), using the Mass Lynx workstation for data analysis. Ions were monitored for at least one transition.

#### 2.6.2. GC-µECD

Samples were injected (1.0 µL) using an Agilent Technologies 7693 Split/spitless autosampler to an Agilent 7890B gas chromatograph with Flame Photometric Detector (FPD) and micro-electron capture detector (µ-ECD) at 300 °C. The column was VF-5 of 30 m × 0.25 mm × 0.25 µm. An oven temperature ramp from 100 to 275 °C was used. Helium and nitrogen were used as mobile phases with a flow of 1.5 mL/min and 30 mL/min, respectively. During the initial stage, the oven temperature was set to 100 °C for a duration of 2 min. It was then increased to 170 °C at a rate of 20 °C/min, lasting for 1.25 min. After that, the temperature was raised to 270 °C at a rate of 4 °C/min, maintained for 12 min, and concluded with a final time of 45 min per run.

### 2.7. Analytical Method Validation

To ensure the quality of the analytical results, the analytical methods used, i.e., UHPLC-MS/MS and GC-µECD, were validated in linearity, precision, and accuracy following the standards described in the Servicio Nacional de Sanidad, Inocuidad y Calidad Agroalimentaria of Mexico (SENASICA) [23]. Moreover, the parameters and the limit of detection (LOD) and quantification (LOQ) were estimated through the guidelines of the U.S. Environmental Protection Agency (EPA) [24].

### 2.8. Degradation, Half-Life, and Permanence of Acetamiprid, Azoxystrobin, and β-Cyfluthrin in Jalapeño Pepper

The dissipation patterns of the pesticides were performed using the Wilhelmey equation of a first-order reaction (Equation (1)). The dissipation constants and determination coefficients (r^2^) were calculated using exponential regression, using the Minitab 17 software (State College, PA, USA). A significant adjustment of ≤0.05 was established. The half-life of the pesticides was calculated using Equation (2), in which the constant *k* is the slope of the exponential regression (1):

C_t_ = C_0_ × e^−*k*t^ (1)

where C_t_ represents the sample residue at time t (mg/kg), C_0_ represents the initial sample residue (mg/kg), and *k* refers to the dissipation rate constant. Note that, in a first-order reaction, *k* is constant, that is, it does not depend on the initial concentration C_0_.

The permanence of the pesticides was calculated with the following Equation (2):

(2) $$t_{1/2}=\frac{Ln2}{k}$$

where *t*_1/2_ = half-life, *k* = dissipation constant of the pesticide (days), *Ln* = natural logarithm.

### 2.9. Experimental Design

A completely randomized one-factor (dissipation time) ANOVA was performed, with seven levels (1 h, 1, 3, 5, 7, and 14 days). Sampling was performed in duplicate, while pesticide quantification was carried out in triplicate. The field experiment was developed in crop plots, which comprised seven double-row rows. The rows were numbered 1 to 7, to which three treatments were applied. The treatments consisted of applying pesticides at intervals of 7 days, and sampling was carried out randomly, where the response variable was the residuality of the pesticides under study.

## 3. Results and Discussion

### 3.1. Method Validation

To conduct the dissipation kinetic studies of the various pesticide molecules, a preliminary study was essential to validate the performance and reliability of the analytical method employed. The QuEChERS extraction method was validated to analyze acetamiprid and azoxystrobin using LC-MS/MS and β-cyfluthrin using GC-µECD in jalapeño pepper. The results are shown in Table 2. All data were considered to be acceptable, and our method was reliable for the determination of the selected pesticides in the jalapeño pepper samples. For instance, the method demonstrated linearity, precision, and accuracy within acceptable reference ranges. Recovery percentages ranged from 92% to 117%, with coefficients of variation below 18%. The limits of detection (LODs) and quantification (LOQ) were determined to be in the ranges of 0.011–0.022 mg/kg and 0.034–0.068 mg/kg, respectively. The experimental data for the determination of LODs and LOQs are depicted in Table S1.

Quantification was performed using a linear least squares calibration, where analyte peak areas were plotted against analyte concentrations. The calibration curve’s ordinate at the origin was required to be close to zero, and the correlation coefficient (R^2^) had to exceed 0.99 (Table S2). As demonstrated, all compounds displayed excellent linearity within concentration ranges from 2.5 to 50 µg/L and 50 to 1000 µg/L for the respective detection systems, with determination coefficients (R^2^) greater than 0.99. The linearity plots are depicted in Figure S1.

For the linear working range, jalapeño pepper matrix blanks were fortified at three concentration levels, ranging from 0.03 to 0.30 mg/kg for each analyte. The relationship between the amount of analyte added and the amount recovered was evaluated. All analytes demonstrated good linearity, with correlation coefficients (r) exceeding 0.98 (Figure S2).

The method proved to be highly efficient for the extraction of the residues of the model analytes. Recovery percentages (%R) ranged from 92% to 117%, with coefficients of variation (%CV) below 18% (Figure S3). According to Lehotay et al. [22], acceptable recovery averages should fall within a range from 70% to 120%, with %CV values ≤ 20%. The results obtained demonstrate that the method is both reproducible and sufficiently sensitive for the determination of acetamiprid, azoxystrobin, and β-cyfluthrin in jalapeño peppers at residue levels below the established MRLs.

### 3.2. Residuality of Acetamiprid, Azoxystrobin, and β-Cyfluthrin in Jalapeño Peppers in a Greenhouse and Open Field

**Figure 2:**
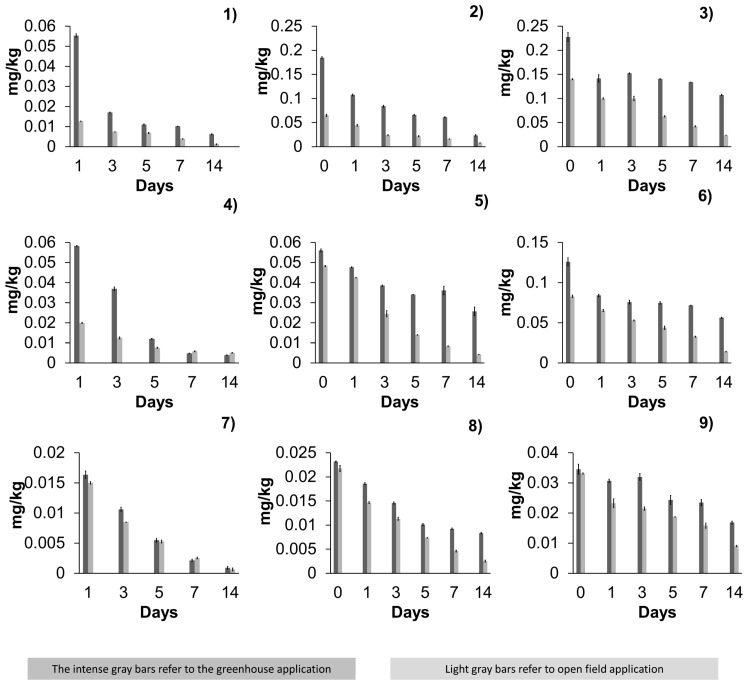
Residues of acetamiprid, azoxystrobin, and β-cyfluthrin in jalapeño pepper. Figure (**1**) depicts the acetamiprid, first application; Figure (**2**) refers to a second application, while Figure (**3**) refers to a third application. In Figure (**4**) azoxystrobin (first application) is depicted. Figure (**5**) depicts the second application, and the third application Figure (**6**). On the other hand, in β-cyfluthrin Figure (**7**) first application was depicted. And Figure (**8**) shows the second application residues. While in Figure (**9**), the third β-cyfluthrin application was displayed. Note that dark grey bars correspond to greenhouse application, and light grey bars correspond to open-field application.

The dissipation pattern of the pesticides differed due to their physicochemical characteristics and modes of action (Table 3). The cultivation systems showed significant differences in the dissipation of the three pesticides studied, with higher quantified residues in greenhouses than in open fields. As the sampling time passed, degradation increased in open-field production. However, the average percentage of dissipation between each application decreased (Figure 2). During the first application, the degradation time is longer, with a tendency to decrease as the applications progress due to the bioaccumulation of pesticides in the plant and the fruits [25].

#### 3.2.1. Residuality of Acetamiprid

Acetamiprid dissipation was higher in the open-field peppers during the last two applications; only during the first application was it higher in the greenhouse peppers. However, the average residual content was higher in the greenhouse peppers in all three applications (Table 3). In both open-field and greenhouse production system applications, a decrease in the residues was observed as the sampling time progressed, mainly in the open-field production system. However, the average dissipation between each application suggests that with more applications, there is a lower degradation percentage.

Similarly, it has been reported that acetamiprid is rapidly degraded in pepper fruits grown in open fields, observing that, after 1 day, the residues dissipated up to 21%, increasing over time and reaching up to 69% on day 7, with a half-life of 4.84 days [26]. On the other hand, another study reports that, in open-field-grown peppers, 99.39% of acetamiprid dissipated by day 15, with a half-life of 2.27 days [27]. Different climatic conditions affect the breakdown of pesticides, making it essential to conduct these studies under actual production conditions in Mexico.

#### 3.2.2. Residuality of Azoxystrobin

In the case of the dissipation of azoxystrobin, the highest concentrations quantified were in greenhouse peppers (Table 3). Dissipation was more significant in the open field during the second and third applications. As the applications progressed, the dissipation percentages decreased in both production systems. Similarly, the dissipation of azoxystrobin in Almuden var. pepper grown in a greenhouse showed an accumulation in the active ingredient in the fruits, reaching 1.14 mg/kg after the second application due to the systemic properties of the pesticide. This behavior explains the decrease in its dissipation as the applications progressed [28]. In other crops grown in open fields, such as cucumber, the degradation of azoxystrobin is slower than other pesticides, such as methyldinocap. However, it is fast enough to dissipate before harvest since its half-life is only 0.9–4.7 days, dissipating 20% after the first day of application [29]. For instance, in greenhouse-grown tomatoes var. Harzfeuer, maximum concentrations of 0.917 ± 0.094 mg/kg have been reported during the first hour after application, with dissipation of 98.36% by day 21 [30].

#### 3.2.3. Residuality of β-Cyfluthrin

Regarding β-cyfluthrin, the maximum average dissipation was observed during the first application in both production systems (Table 3). During the second application, the percentage of dissipation was higher in open-field peppers, and in the third application, the dissipation was higher in the greenhouse. It was observed that as the applications progressed, the % of dissipation decreased in both production systems. Little has been studied about the dissipation of β-cyfluthrin in pepper fruits. For instance, in hot pepper var. Kranti grown in open fields, it was reported that the residues of β-cyfluthrin on day 10 of sampling decreased below the LOQ (0.05 mg/kg), observing a behavior similar to that reported in our study [31]. The dissipation of β-cyfluthrin in other crops, such as eggplants grown in open fields, reached 92.31% on day 5 after the third application; these results suggest the rapid degradation of β-cyfluthrin [32].

In all graphs, dark grey bars represent residues from greenhouse applications, while light grey bars represent residues from open-field applications. The figure illustrates the dissipation patterns of each pesticide across multiple applications, highlighting the differences between greenhouse and open-field conditions.

### 3.3. Climatic Conditions and Their Relationship with the Dissipation of Acetamiprid, Azoxystrobin, and β-Cyfluthrin in Jalapeño Pepper

During the dissipation experiment, climatic conditions were monitored to elucidate their relationship with pesticide degradation (Table 4). Evapotranspiration (ET_0_) is a physical process that encompasses two interconnected phenomena: the evaporation of water from the soil and plant surfaces, and the transpiration of water through the leaves of plants. The factors that intervene in ET_0_ are solar radiation, relative humidity, temperature, wind speed, and vapor pressure, closely related to pesticide degradation [33,34]. According to our results, the ET_0_ was higher in open fields (2.2 mm), while in greenhouses, it was 12% lower (Table 4). The relationship between the ET_0_ and the dissipation of the pesticides studied suggests a direct influence between both. This is due to the light radiation, temperature, relative humidity, and wind that are considered degrading factors, in addition to the fact that changes in these parameters modify the metabolism of pesticides in plants and, therefore, their absorption and degradation speeds are modified [4,25].

Regarding acetamiprid (Figure S4), a higher accumulated ET_0_ was observed in the open-field peppers during the first application. However, the accrued dissipation in the greenhouse peppers increased as the sampling days passed. During the second application, the accumulated ET_0_ and the % of accumulated dissipation were higher in the open-field samples.

In azoxystrobin (Figure S5), it was observed that, during the first application, the dissipation was higher in the greenhouse. At the same time, the ET_0_ was higher in the open-field samples, and the dissipation during the second and third applications was higher in the open-field samples, observing a similar accumulated ET_0_ between both production systems.

The dissipation and accumulated ET_0_ of β-cyfluthrin (Figure S6) differed between the two production systems. In the first two applications, the % of accumulated dissipation was higher in the open-field samples; meanwhile, in the third application, it was higher in the greenhouse. The same trend was observed for the accumulated ET_0_. These results suggest a direct correlation between the accumulated ET_0_ and the degradation of the pesticide.

Our results agree with previous studies, which mention that the dissipation of acephate, quinalphos, triazophos, and chlorpyriphos in pepper is greater in open fields than in greenhouses because, in greenhouses, there is very low volatilization [35]. Likewise, the increased dissipation of flubendiamide, thiamethoxam, acephate, and methamidophos has been reported in peppers grown in open fields than in greenhouses, relating to climatic conditions such as rain, relative humidity, solar exposure, and temperature to their degradation [36,37,38]. The initial deposition, persistence, and half-life of tricyclazole and mancozeb in peppers grown in sites with less rainfall and humidity are more significant because plants with higher humidity absorb the active ingredients better, facilitating penetration and movement within the plant [39]. Factors such as evaporation and photodegradation also influence the dissipation of pesticides such as azoxystrobin, pyrimethanil, cyprodinil, and fludioxonil [40].

### 3.4. Dissipation Kinetics of Acetamiprid, Azoxystrobin, and β-Cyfluthrin in Jalapeño Pepper in a Greenhouse and Open Field

**Figure 3:**
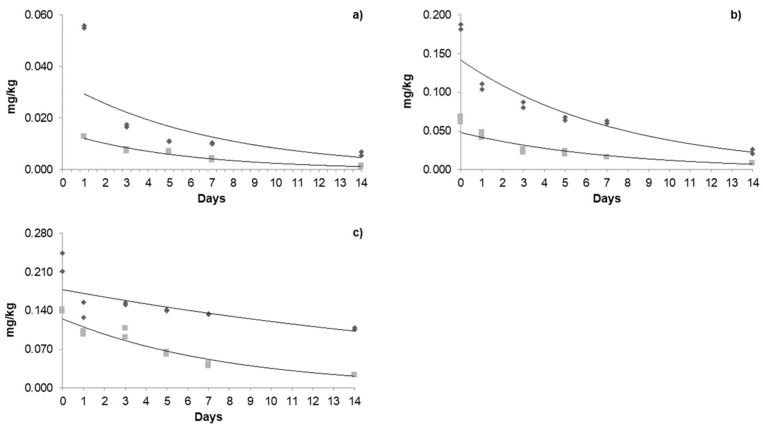
Dissipation kinetics of acetamiprid in the greenhouse and open field: (**a**) first application; (**b**) second application; (**c**) third application. The dark grey box and line refers to greenhouse kinetics, and the light grey box and line refers to open-field kinetics.

**Figure 4:**
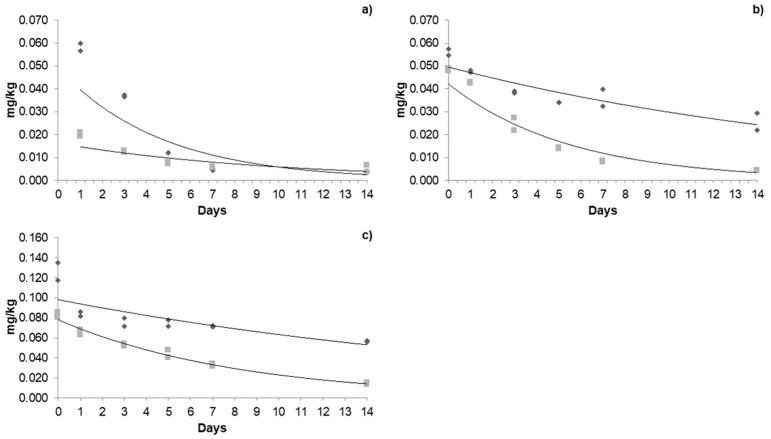
Dissipation kinetics of azoxystrobin in greenhouse and open field: (**a**) first application; (**b**) second application; (**c**) third application. The dark grey box and line refers to the kinetics in a greenhouse, and the light grey box and line refers to open-field kinetics.

**Figure 5:**
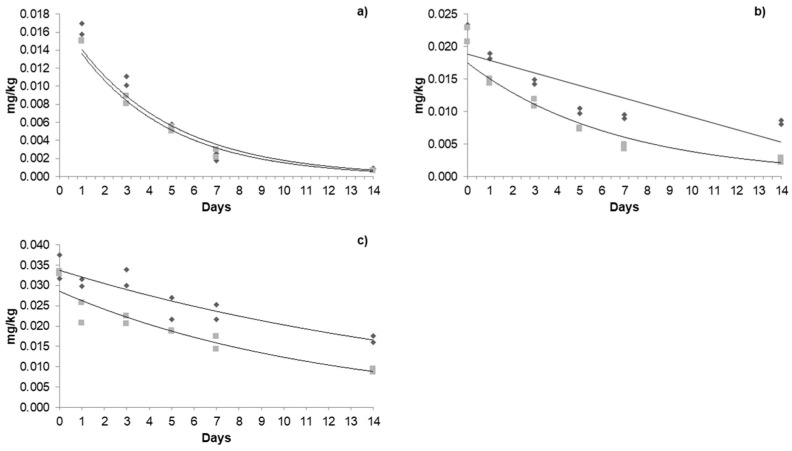
Dissipation kinetics of β-cyfluthrin in a greenhouse and open-field: (**a**) first application; (**b**) second application; (**c**) third application. The dark grey box and line refers to greenhouse kinetics, and the light grey box and line refers to open-field kinetics.

The dissipation of acetamiprid (Figure 3), azoxystrobin (Figure 4), and β-cyfluthrin (Figure 5) followed first-order kinetics under both types of cultivation. The exponential mathematical model provided the best fit for first-order dissipation kinetics, consistent with findings reported by Galietta et al. [41] for acetamiprid in peaches and Malhat et al. (2016) [42] for lambda-cyhalothrin in open-field tomatoes.

For instance, the persistence of pesticides is generally expressed in terms of half-life (*t*_1/2_), i.e., time for disappearance of pesticide to 50 percent of its initial concentration, which could be expressed using a first-order equation (see Equation (3)), in which the constant *k* is the slope of the linear regression. In general, the highest concentrations were recorded on the day of application for the three pesticides; however, the residues decreased exponentially over time. In addition to degradation due to climatic conditions, the fruits, when exposed to the active ingredients, begin a detoxification and metabolization process through the chemical reactions of oxidation, reduction, hydrolysis, and conjugation to glycosides, which are metabolized more quickly [25,43].

#### 3.4.1. Dissipation Kinetics of Acetamiprid in a Greenhouse and Open Field

Regarding the dissipation behavior of acetamiprid (Table 5) in the greenhouse, the initial residues of the three applications were 0.055, 0.185, and 0.227 mg/kg; a cumulative effect was observed between applications, with the % of dissipation decreasing mainly in the greenhouse. In the open field, the initial residues were 0.013, 0.065, and 0.140 mg/kg. As the days passed, the degradation became increasingly noticeable; on day 14, only 0.001, 0.008, and 0.024 mg/kg were quantified. Note that these concentrations were below the MRLs. The % of dissipation during the third application on day 14 was 83% for the open field and 55% for the greenhouse. In this sense, the application and dosage intervals play an important role in the deposition and degradation of the pesticides studied. The half-life (*t*_1/2_) of acetamiprid in the greenhouse was 2.15, 7.02, and 14.65 days for each application, respectively, with a constant increase observed between applications. This may be due to the systemic accumulation of pesticides. In the open field, the half-life for each application was 4.5, 6.42, and 4.78 days. The residence time in the greenhouse for each application was 10.2, 22, and 47 days; moreover, for the open field, they were 11.5, 14.7, and 28 days, respectively. The different residence times, half-life, and the degradation of acetamiprid in the greenhouse and open fields may be due to differences in their exposure to ultraviolet light and light intensity since this pesticide is susceptible to photolysis [44,45]. These parameters may vary depending on the doses and application intervals, type of fruit, and climatic conditions. For instance, the half-life of acetamiprid in cowpea fruits was 2.34 days [46], 2.68 days in parsley leaves [47], and 5.2 days in kimchi cabbages [48]; moreover, in greenhouse-grown tomatoes, it is 2.07 days [49], similar to that reported in our investigation.

#### 3.4.2. Dissipation Kinetics of Azoxystrobin in a Greenhouse and Open Field

Regarding azoxystrobin, the initial concentrations of each application in the greenhouse were 0.085, 0.056, and 0.126 mg/kg; none of these concentrations exceeded the MRL. In the open field, the concentrations were 0.020, 0.048, and 0.083 mg/kg. A constant increase was observed as the applications progressed. The half-life in the greenhouse was 2.73, 11.96, and 12.55 days; meanwhile, in the open field, they were 4.46, 3.01, and 5.48 days. Residence times in the greenhouse were up to 58.9 days; in the open field, it was up to 23 days. Azoxystrobin degradation is mainly due to photolysis and hydrolysis [50], in addition to the difference in climatic factors between each production system. The half-life of azoxystrobin has been reported in other crops, such as in tomatoes grown in greenhouses, which is 3.55 days [30], and in dragon fruit, three days [51]. In greenhouse-grown peppers, 15.21 days were observed, and a decrease was observed during the second application (13.54 days) [28].

#### 3.4.3. Dissipation Kinetics of β-Cyfluthrin in a Greenhouse and Open Field

The initial residues of β-cyfluthrin in the greenhouse during the three applications were 0.016, 0.023, and 0.035 mg/kg. A cumulative effect was observed in the last two; however, the concentrations were detected below the MRL. In the open field, the initial residues were 0.015, 0.022, and 0.033 mg/kg. The half-life in the greenhouse ranged between 3.21 and 12.55 days, while, in the open field, it was 3.18 and 7.46 days. The residence time in the greenhouse was 6.6 to 39 days, and, in the open field, it was 6 to 21 days from the first to the third application (Table 5). Other studies mention that the half-life of β-cyfluthrin in pepper is 3.54 days at the recommended doses, which is consistent with our results [31]. In other fruits, such as okra, the half-life of β-cyfluthrin was 0.68 days at doses of 36 g a.i. ha [52].

To ensure that pesticide residues remain below established tolerance thresholds and that edible parts are safe for human consumption, it is crucial to monitor pesticide residues on vegetable and fruit crops post-application. This monitoring helps determine the appropriate pre-harvest interval (PHI) and the latency period between pesticide application and harvest.

## 4. Conclusions

The dissipation patterns of acetamiprid, azoxystrobin, and β-cyfluthrin in jalapeño pepper differed between open-field and greenhouse conditions when applied at recommended doses. Initial residue concentrations, half-lives, and residence times for all three pesticides were higher in the greenhouse compared to the open field. The half-life and persistence of the pesticides were lower during the initial applications but increased over time with subsequent applications. Dissipation occurred more rapidly in the open field than in the greenhouse. Notably, residue concentrations detected during the third application were below the MRLs, indicating no significant health risks. These findings underscore the importance of determining appropriate latency periods and further investigating dissipation dynamics to ensure that pesticide residues degrade to safe levels before commercialization and consumption.

## Figures and Tables

**Table 1:** Dosage and characteristics of acetamiprid, azoxystrobin, and β-cyfluthrin.

Commercial Name	Active Ingredient	Dosage	a.i/ha (g)	Safety Interval (Days)	Vapor Pressure (mPa)	ADI * (mg/kg)	MRL * (mg/L)
Bulldock 125^®^ (Bayer^®^)	β-cyfluthrin (5%)	100 cm^3^/ha	5	5	3 × 10^−4^	0.02	0.5
Rescate 20 PS^®^ (SummitAgro^®^)	Acetamiprid (20%)	250 g/ha	40	7	1.73 × 10^−4^	0.07	1
Amistar^®^ (Syngenta^®^)	Azoxystrobin (50%)	115 g/ha	57.5	3	1.1 × 10^−7^	0.2	3

ADI (acceptable daily intake). MRL (maximum residue limit). a.i/ha (active ingredient per ha). * Values established using the USDA.

**Table 2:** Performance parameters of the analytical method developed.

Pesticide	Recovery (%R)	Accuracy Under Repeatability Conditions (%CV)	Limit of Detection (mg/kg)	Limit of Quantitation (mg/kg)	Linearity (R^2^)
Acetamiprid	106.7 ± 1.6	17.99	0.022	0.068	0.998
Azoxystrobin	104.3 ± 6.8	6.88	0.011	0.034	0.997
β-cyfluthrin	109.9 ± 7.7	8.30	0.013	0.039	0.996
Criteria	70–120%	CV ≤ 20%			R^2^ ≥ 0.99

**Table 3:** Residuality (mg/kg) and percentage of dissipation (D (%)) of acetamiprid, azoxystrobin, and β-cyfluthrin in jalapeño peppers grown in the greenhouse and open-field production systems.

Pesticide	Days	First Application				Second Application				Third Application			
		Greenhouse		Open Field		Greenhouse		Open Field		Greenhouse		Open Field	
		mg/kg	D (%)	mg/kg	D (%)	mg/kg	D (%)	mg/kg	D (%)	mg/kg	D (%)	mg/kg	D (%)
Acetamiprid	0					0.185	0	0.065	0	0.227	0	0.140	0
	1	0.055	0	0.013	0	0.107	39	0.044	41	0.141	48	0.100	30
	3	0.017	70	0.007	45	0.084	52	0.024	68	0.152	37	0.100	22
	5	0.011	80	0.007	44	0.066	65	0.022	66	0.141	43	0.063	56
	7	0.010	82	0.004	67	0.061	67	0.016	77	0.134	45	0.042	68
	14	0.006	88	0.001	89	0.023	89	0.008	88	0.107	55	0.024	83
Azoxystrobin	0					0.056	0	0.048	0	0.126	0	0.083	0
	1	0.058	0	0.020	0	0.048	16	0.042	12	0.084	39	0.065	16
	3	0.037	35	0.013	42	0.039	32	0.024	55	0.076	47	0.053	36
	5	0.012	79	0.008	61	0.034	41	0.014	71	0.075	47	0.044	50
	7	0.005	91	0.006	71	0.036	31	0.008	82	0.072	47	0.033	58
	14	0.004	94	0.005	69	0.026	62	0.004	91	0.056	59	0.014	81
β-cyfluthrin	0					0.023	0	0.022	0	0.035	0	0.033	0
	1	0.016	0	0.015	0	0.019	19	0.015	28	0.031	34	0.023	38
	3	0.011	34	0.009	46	0.015	39	0.011	43	0.032	48	0.022	33
	5	0.005	66	0.005	63	0.010	58	0.007	64	0.024	67	0.019	44
	7	0.002	90	0.003	80	0.009	62	0.005	79	0.023	81	0.016	48
	14	0.001	95	0.001	96	0.008	65	0.003	89	0.017	90	0.009	72

**Table 4:** Weather conditions during pesticide application in jalapeño pepper.

Climatic Factors	Greenhouse	Open Field
Maximum temperature (°C)	38.77	32.8
Minimum temperature (°C)	5.6	5.3
Mean temperature (°C)	28	23.3
Mean luminous intensity (Watts/m^2^)	640	721
Mean relative humidity (%)	77	76
Mean ET_0_ (mm)	2.2	2.5

**Table 5:** Evaluation parameters in the dissipation kinetics of acetamiprid, azoxystrobin, and β-cyfluthrin in jalapeño pepper grown in a greenhouse and open field.

Pesticide	Cultivation Type	First Application			Second Application			Third Application		
		R_0_ (mg/kg)	*t*_(1/2)_ (Days)	Pr (Days)	R_0_ (mg/kg)	*t*_(1/2)_ (Days)	Pr (Days)	R_0_ (mg/kg)	*t*_(1/2)_ (Days)	Pr (Days)
Acetamiprid	Greenhouse	0.055	2.15	10.2	0.185	7.02	22	0.227	14.65	47
	Open field	0.013	4.50	11.5	0.065	6.42	14.7	0.140	4.78	28
Azoxystrobin	Greenhouse	0.058	2.73	11.8	0.056	11.96	43.5	0.126	12.55	58.9
	Open field	0.020	4.46	12	0.048	3.01	11.7	0.083	5.48	23
β-cyfluthrin	Greenhouse	0.016	3.21	6.6	0.023	6.42	15.4	0.035	12.55	39
	Open field	0.015	3.18	6	0.022	2.91	7.8	0.033	7.46	21

R_0_ = Initial residues, *t*_1/2_ = Half-life, Pr = Persistence.

## Data Availability

The original contributions presented in this study are included in the article/Supplementary Materials. Further inquiries can be directed to the corresponding authors.

## Supplementary Materials

The following supporting information can be downloaded at: https://www.mdpi.com/article/10.3390/foods14061023/s1, Table S1. Limit of detection (LOD) and limit of quantitation (LOQ). Table S2. Linearity found within the range of 5 to 100 µg/L and 50 to 1000 µg/L. Figure S1. Linearity plots of the system for the model analytes. (A) Acetamiprid. (B) azoxystrobin. (C) β-cyfluthrin. Figure S2. Linear working range for the pesticide molecules (A) acetamiprid. (B) azoxystrobin. (C). β-cyfluthrin. Figure S3. Recovery percentages for acetamiprid, azoxystrobin, and β-cyfluthrin. Figure S4. Cumulative ET0 and cumulative dissipation of acetamiprid in jalapeno pepper in the greenhouse and open field. (a) Greenhouse application (1) first application, (2) second application, (3) third application. (b) Application in open field (1) first application, (2) second application, (3) third application. The dark gray bar refers to the accumulated ET0, and the light gray bar refers to the percentage of acetamiprid residue. Figure S5. Cumulative ET0 and cumulative dissipation of azoxystrobin in jalapeno pepper in greenhouse and open field. (a) Greenhouse application (1) first application, (2) second application, (3) third application. (b) Application in open field (1) first application, (2) second application, (3) third application. The dark gray bar refers to the accumulated ET0, and the light gray bar refers to the percentage of azoxystrobin residue. Figure S6. Cumulative ET0 and cumulative dissipation of β-cyfluthrin in jalapeno pepper in greenhouse and open. (a) Greenhouse application (1) first application, (2) second application, (3) third application. (b) Application in open field (1) first application, (2) second application, (3) third application. The dark gray bar refers to the accumulated ET0, and the light gray bar refers to the percentage of β-cyfluthrin residue.

## References

[1] Cooper J., Dobson H. (2007). The benefits of pesticides to mankind and the environment. Crop Prot..

[2] Souza M.C.O., Cruz J.C., Cesila C.A., Gonzalez N., Rocha B.A., Adeyemi J.A., Nadal M., Domingo J.L., Barbosa F. (2023). Recent trends in pesticides in crops: A critical review of the duality of risks-benefits and the Brazilian legislation issue. Environ. Res..

[3] Food and Agriculture Organization/World Health Organization (2023). Maximum Residue Limits (MRL). https://www.fao.org/fao-who-codexalimentarius/codex-texts/maximum-residue-limits/es/#:~:text=Un%20l%C3%ADmite%20m%C3%A1ximo%20de%20residuos,a%20las%20buenas%20pr%C3%A1cticas%20agr%C3%ADcolas.

[4] Fantke P., Juraske R. (2013). Variability of Pesticide Dissipation Half-Lives in Plants. Environ. Sci. Technol..

[5] Shim J.-H., Eun J.-B., Zaky A.A., Hussein A.S., Hacimüftüoğlu A., Abd El-Aty A.M. (2023). A Comprehensive Review of Pesticide Residues in Peppers. Foods.

[6] Food and Agriculture Organization of the United Nations (2023). Food and Agriculture Data. https://www.fao.org/faostat/en/#home.

[7] SAGARPA (2017). Planeación Agrícola Nacional. Chiles y Pimientos Mexicanos. https://www.gob.mx/cms/uploads/attachment/file/257072/Potencial-Chiles_y_Pimientos-parte_uno.pdf.

[8] SIAP (2024). Anuario Estadístico de la Producción Agrícola. https://www.gob.mx/siap/acciones-y-programas/produccion-agricola-33119.

[9] Faraji M., Noorbakhsh R., Shafieyan H., Ramezani M. (2018). Determination of acetamiprid, imidacloprid, and spirotetramat and their relevant metabolites in pistachio using modified QuEChERS combined with liquid chromatography-tandem mass spectrometry. Food Chem..

[10] Majumder S., Mandal S., Majumder B., Paul A., Paul T., Sahana N., Mondal P. (2022). A liquid chromatographic method for determination of acetamiprid and buprofezin residues and their dissipation kinetics in paddy matrices and soil. Environ. Sci. Pollut. Res..

[11] Abdelraheem E.M.H., Hassan S.M., Arief M.M.H., Mohammad S.G. (2015). Validation of quantitative method for azoxystrobin residues in green beans and peas. Food Chem..

[12] Gautam M., Fomsgaard I.S. (2017). Liquid chromatography-tandem mass spectrometry method for simultaneous quantification of azoxystrobin and its metabolites, azoxystrobin free acid and 2-hydroxybenzonitrile, in greenhouse-grown lettuce. Food Addit. Contam. Part A.

[13] Mukherjee S., Mukherjee S., Das G.K., Bhattacharyya A. (2015). Analytical method validation and comparison of two extraction techniques for screening of azoxystrobin from widely used crops using LC–MS/MS. J. Food Meas. Charact..

[14] Laskowski D.A., Ware G.W. (2002). Physical and Chemical Properties of Pyrethroids. Reviews of Environmental Contamination and Toxicology: Continuation of Residue Reviews.

[15] Chawla S., Shah P.G., Patel A.R., Patel H.K., Vaghela K.M., Solanki P.P. (2018). Residue determination of β-cyfluthrin and imidacloprid as mix formulation in/on chickpea (Cicer arietinum) pods and soil and its risk assessment. Food Qual. Saf..

[16] Liu W., Gan J.J. (2004). Separation and Analysis of Diastereomers and Enantiomers of Cypermethrin and Cyfluthrin by Gas Chromatography. J. Agric. Food Chem..

[17] You J., Wang D., Lydy M.J. (2010). Determination of pyrethroid insecticides in sediment by gas chromatography—Ion trap tandem mass spectrometry. Talanta.

[18] European Food Safety A., Arena M., Auteri D., Brancato A., Bura L., Carrasco Cabrera L., Chiusolo A., Court Marques D., Crivellente F., De Lentdecker C. (2020). Peer review of the pesticide risk assessment of the active substance beta-cyfluthrin. EFSA J..

[19] Oliveira L.G.d., Kurz M.H.S., Guimarães M.C.M., Martins M.L., Prestes O.D., Zanella R., Ribeiro J.N.d.S., Gonçalves F.F. (2019). Development and validation of a method for the analysis of pyrethroid residues in fish using GC–MS. Food Chem..

[20] Tian F., Qiao C., Luo J., Guo L., Pang T., Pang R., Li J., Wang C., Wang R., Xie H. (2020). Method development and validation of ten pyrethroid insecticides in edible mushrooms by Modified QuEChERS and gas chromatography-tandem mass spectrometry. Sci. Rep..

[21] Steiner A.A. (1961). A universal method for preparing nutrient solutions of a certain desired composition. Plant Soil.

[22] Lehotay S.J. (2007). Determination of Pesticide Residues in Foods by Acetonitrile Extraction and Partitioning with Magnesium Sulfate: Collaborative Study. J. Aoac Int..

[23] SENASICA (2022). GÚIA DE VALIDACIÓN DE MÉTODOS PARA EL ANÁLISIS DE PLAGUICIDAS. https://www.gob.mx/cms/uploads/attachment/file/702365/SMEC-PR-GVP_G_IA_DE_VALIDACI_N_DE_M_TODOS_PARA_EL_AN_LISIS_DE_PLAGUICIDAS__1_.pdf.

[24] EPA (2000). ASSIGNING VALUES TO NONDETECTED/NON-QUANTIFIED PESTICIDE RESIDUES IN HUMAN HEALTH FOOD EXPOSURE ASSESSMENTS. https://archive.epa.gov/pesticides/trac/web/pdf/trac3b012.pdf.

[25] Zhang J.J., Yang H. (2021). Metabolism and detoxification of pesticides in plants. Sci. Total Environ..

[26] Sanyal D., Chakma D., Alam S. (2008). Persistence of a Neonicotinoid Insecticide, Acetamiprid on Chili (*Capsicum annum* L.). Bull. Environ. Contam. Toxicol..

[27] Varghese T.S., Mathew T.B., George T., Beevi S.N., Xavier G. (2015). Persistence and dissipation of neonicotinoid insecticides on chilli fruits. Qual. Assur. Saf. Crops Foods.

[28] Fenoll J., Ruiz E., Hellín P., Lacasa A., Flores P. (2009). Strobilurin residue levels in greenhouse-grown pepper and under cold-storage conditions. J. Sci. Food Agric..

[29] Bian Y., Guo G., Liu F., Chen X., Wang Z., Hou T. (2020). Meptyldinocap and azoxystrobin residue behaviors in different ecosystems under open field conditions and distribution on processed cucumber. J. Sci. Food Agric..

[30] Jankowska M., Kaczynski P., Hrynko I., Lozowicka B. (2016). Dissipation of six fungicides in greenhouse-grown tomatoes with processing and health risk. Environ. Sci. Pollut. Res..

[31] Ahlawat S., Chauhan R., Malik K., Yadav S.S., Kumari N. (2021). Persistence and processing effects in reduction of residues of β-cyfluthrin + imidacloprid and its metabolite in hot pepper. Int. J. Environ. Anal. Chem..

[32] Mandal K., Chahil G.S., Sahoo S.K., Battu R.S., Singh B. (2010). Dissipation Kinetics of β-Cyfluthrin and Imidacloprid in Brinjal and Soil Under Subtropical Conditions of Punjab, India. Bull. Environ. Contam. Toxicol..

[33] Jiang S., Xinguang W., Pei D., Zheng S., Fu S., Wang T. (2022). Effective Method of Estimating the Daily Evapotranspiration of Greenhouse Grapes in the Cold Area of Northeast China. ACS Omega.

[34] Yao M., Gao M., Wang J., Li B., Mao L., Zhao M., Xu Z., Niu H., Wang T., Sun L. (2023). Estimating Evapotranspiration of Greenhouse Tomato under Different Irrigation Levels Using a Modified Dual Crop Coefficient Model in Northeast China. Agriculture.

[35] Shukla V.R., Parmar K., Vaghela K.M., Patel J., Chawla S., Patel A.R., Upadhyay P., Shah P., Pathan F.K. (2016). Persistence of Pesticides in Capsicum (*Capsicum annuum* L.) under Greenhouse and Open Field. Pestic. Res. J..

[36] Pathipati V., Singh T.V.K., Vemuri S.B., Reddy R.V.S.K., Bharathi N.B. (2017). Dissipation Dynamics of flubendiamideon Capsicum in Open Field and Poly House Conditions. Int. J. For. Hortic..

[37] Pathipati V.L., Singh T.V.K., Vemuri S.B., Reddy R.V.S.K., Bharathi N.B., Reddy N.R., Aruna (2018). Dissipation Studies of Thiamethoxam on Capsicum under Field and Poly House Conditions. Int. J. Curr. Microbiol. Appl. Sci..

[38] Sharma D., Hebbar S.S., Divakara J.V., Mohapatra S. (2012). Residues of pesticides acephate and methamidophos in capsicum grown in greenhouse and open field. Qual. Assur. Saf. Crops Foods.

[39] Mandal S., Poi R., Hazra D.K., Bhattacharyya S., Banerjee H., Karmakar R. (2022). Assessment of variable agroclimatic impact on dissipation kinetics of ready-mix fungicide formulation in green chili for harmonization of food safety. J. Food Compos. Anal..

[40] Garau V.L., Angioni A., Del Real A.A., Russo M., Cabras P. (2002). Disappearance of Azoxystrobin, Pyrimethanil, Cyprodinil, and Fludioxonil on Tomatoes in a Greenhouse. J. Agric. Food Chem..

[41] Galietta G., Egaña E., Gemelli F., Maeso D., Casco N., Conde P., Nuñez S. (2010). Pesticide dissipation curves in peach, pear and tomato crops in Uruguay*. J. Environ. Sci. Health Part B.

[42] Malhat F., Loutfy N.M., Ahmed M.T. (2016). Dissipation pattern and risk assessment of the synthetic pyrethroid Lambda-cyhalothrin applied on tomatoes under dryland conditions, a case study. Int. J. Food Contam..

[43] Sharma A., Kumar V., Kohli S.K., Kaur R., Kaur T., Arora S., Thukral A.K., Bhardwaj R. (2020). Pesticide Metabolism in Plants, Insects, Soil Microbes and Fishes. Pesticides in Crop Production.

[44] Gupta S., Gajbhiye V.T., Gupta R.K. (2008). Effect of Light on the Degradation of Two Neonicotinoids viz Acetamiprid and Thiacloprid in Soil. Bull. Environ. Contam. Toxicol..

[45] Nicol E., Varga Z., Vujovic S., Bouchonnet S. (2020). Laboratory scale UV–visible degradation of acetamiprid in aqueous marketed mixtures—Structural elucidation of photoproducts and toxicological consequences. Chemosphere.

[46] Fu D., Zhang S., Wang M., Liang X., Xie Y., Zhang Y., Zhang C. (2020). Dissipation behavior, residue distribution and dietary risk assessment of cyromazine, acetamiprid and their mixture in cowpea and cowpea field soil. J. Sci. Food Agric..

[47] Abdallah O., Abdel Ghani S., Hrouzková S. (2017). Development of validated LC-MS/MS method for imidacloprid and acetamiprid in parsley and rocket and evaluation of their dissipation dynamics. J. Liq. Chromatogr. Relat. Technol..

[48] Lee J., Kim B.J., Kim E., Kim J.-H. (2019). Dissipation Kinetics and the Pre-Harvest Residue Limits of Acetamiprid and Chlorantraniliprole in Kimchi Cabbage Using Ultra-Performance Liquid Chromatography-Tandem Mass Spectrometry. Molecules.

[49] Badawy M.E.I., Ismail A.M.E., Ibrahim A.I.H. (2019). Quantitative analysis of acetamiprid and imidacloprid residues in tomato fruits under greenhouse conditions. J. Environ. Sci. Health Part B.

[50] Feng Y., Huang Y., Zhan H., Bhatt P., Chen S. (2020). An Overview of Strobilurin Fungicide Degradation:Current Status and Future Perspective. Front. Microbiol..

[51] Noegrohati S., Sulasmi S., Hernadi E., Asviastuti S. (2019). Dissipation pattern of azoxystrobin and difenoconazole in red dragon fruit (*Hylocereus polyrhizus*) cultivated in Indonesian highland (West Java) and coastal area (D.I. Jogyakarta) and its implication for dietary risk assessment. Food Qual. Saf..

[52] Sahoo S.K., Chahil G.S., Mandal K., Battu R.S., Singh B. (2012). Estimation of β-cyfluthrin and imidacloprid in okra fruits and soil by chromatography techniques. J. Environ. Sci. Health Part B.

