# Supplementary material for: Dynamics of the Dissipation of Acetamiprid, Azoxystrobin, and β-Cyfluthrin in Jalapeño Pepper (Capsicum annuum L.) Produced Under Greenhouse and Open-Field Conditions
Source: Foods. 2025 Mar 17;14(6):1023. doi: 10.3390/foods14061023 (PMC11941646; doi:10.3390/foods14061023)
Supplement: Supplementary file 1 [file foods-14-01023-s001.zip › foods-3511566-supplementary.pdf]

**Supplementary materials**

**Table S1.** Limit of detection (LOD) and limit of quantitation (LOQ)

| **Samples** | **Acetamiprid mg/kg recovered** | **Azoxystrobin mg/kg recovered** | **β-Cyfluthrin mg/kg recovered** |
|---|---|---|---|
| 1 | 0.0359 | 0.0648 | 0.0550 |
| 2 | 0.0353 | 0.0528 | 0.0539 |
| 3 | 0.0373 | 0.0547 | 0.0620 |
| 4 | 0.0542 | 0.0597 | 0.0569 |
| 5 | 0.0527 | 0.0561 | 0.0572 |
| 6 | 0.0553 | 0.0548 | 0.0558 |

| | | | |
|---|---|---|---|
| 7 | 0.0427 | 0.0614 | 0.0475 |
| 8 | 0.0421 | 0.0216 | 0.0491 |
| 9 | 0.0424 | 0.0595 | 0.0509 |
| **Mean** | 0.0442 | 0.0584 | 0.0543 |
| **Standard deviation (SD)** | 0.0079 | 0.0040 | 0.0045 |
| **Coefficient of variation % CV** | 17.888 | 6.8985 | 8.2981 |
| **LOD** | 0.022 | 0.011 | 0.013 |
| **LOQ** | 0.068 | 0.034 | 0.039 |

LOD= t0.99*SD (t0.99 = 2.8965; value from one-tailed Student's t tables with 8 degrees of freedom (n=9), and 99% confidence level). LOQ= LOD*3

**Table S2.** Linearity found within the range of 5 to 100 µg/L and 50 to 1000 µg/L

| Analyte | R.T | Range (µg/L) | Equation (y = bx + a) | $R^2$ |
|---|---|---|---|---|
| Acetamiprid | 2.90 | 5.43 – 106.00 | y= 17074.4x + -11449.2 | 0.9984 |
| Azoxystrobin | 4.99 | 5.43 – 106.00 | y= 45349x + -5311.56 | 0.9976 |
| β-Cyfluthrin | 31.01 | 54.3 – 1080.0 | y= 12715.9x + -135.26 | 0.9988 |

R.T (Retention time).

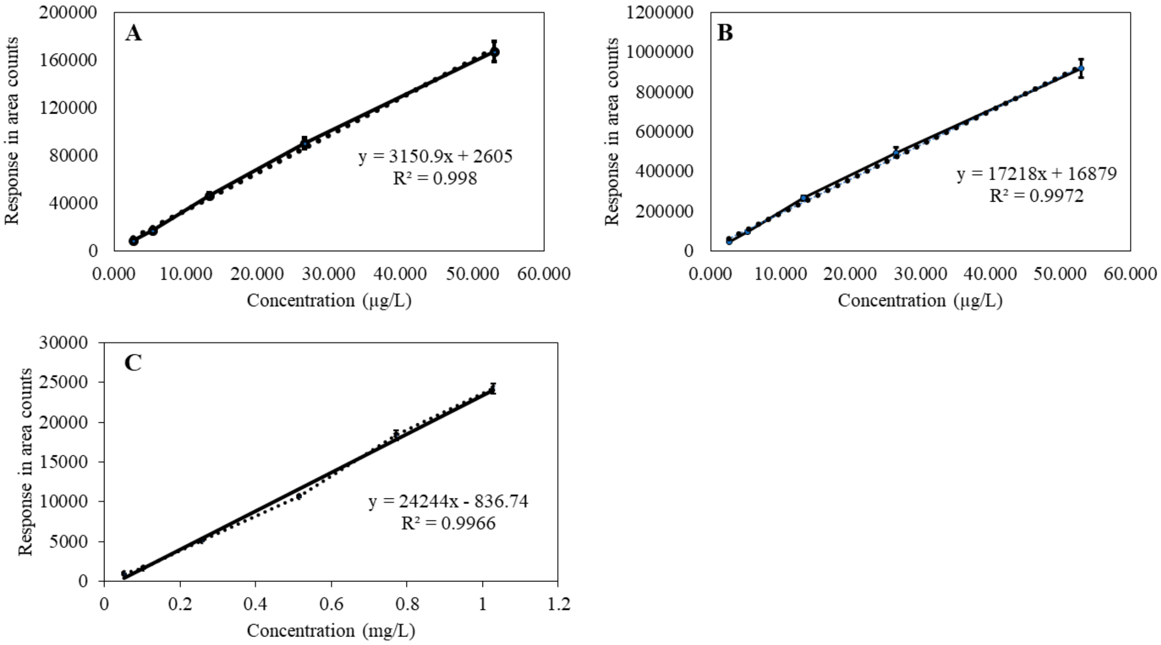


**Figure S1.** Linearity plots of the system for the model analytes. A) Acetamiprid. B) azoxystrobin. C) β-cyfluthrin

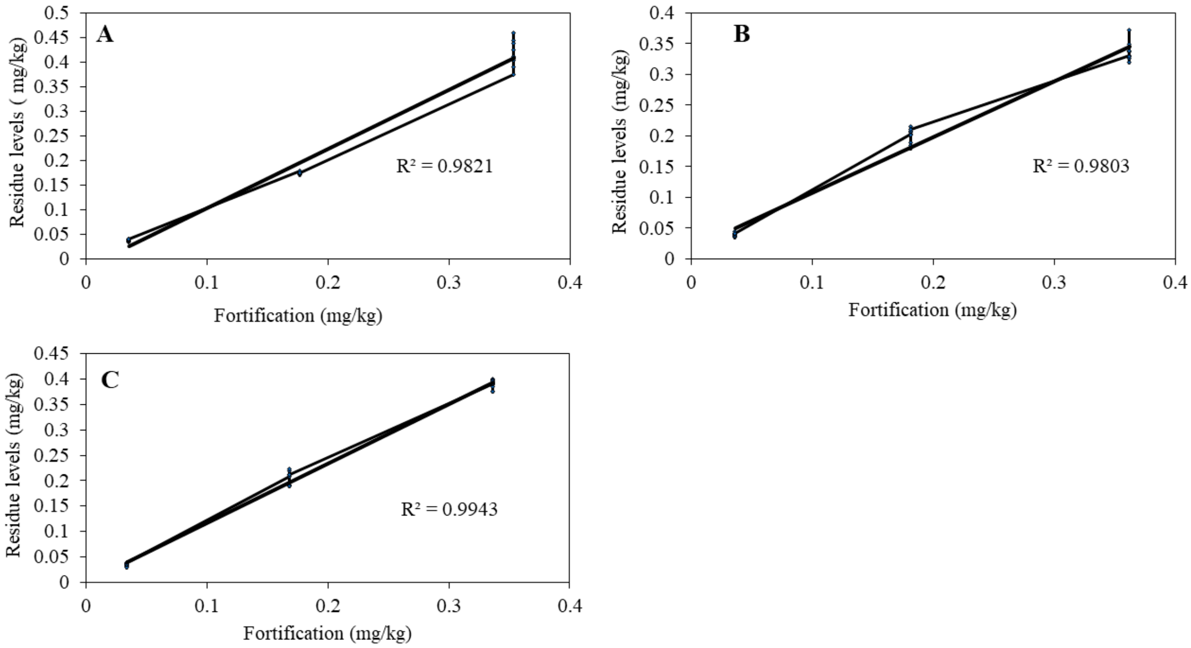


**Figure S2.** Linear working range for the pesticide molecules A) acetamiprid. B) azoxystrobin. C). β-cyfluthrin

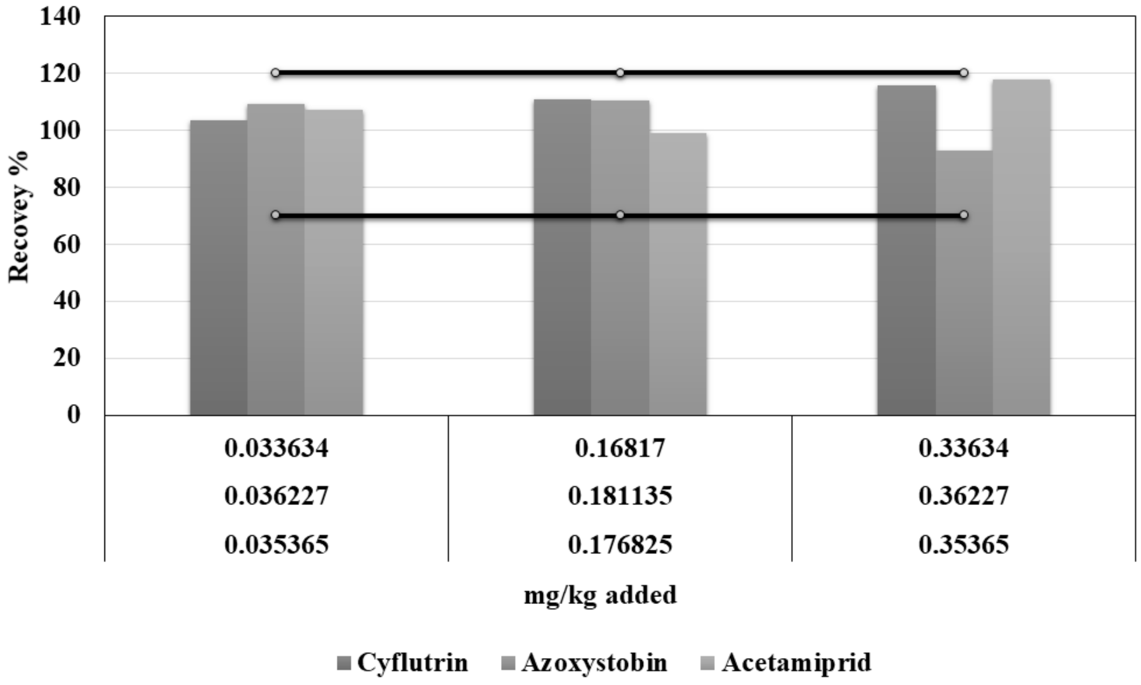


**Figure S3.** Recovery percentages for acetamiprid, azoxystrobin, and β-cyfluthrin

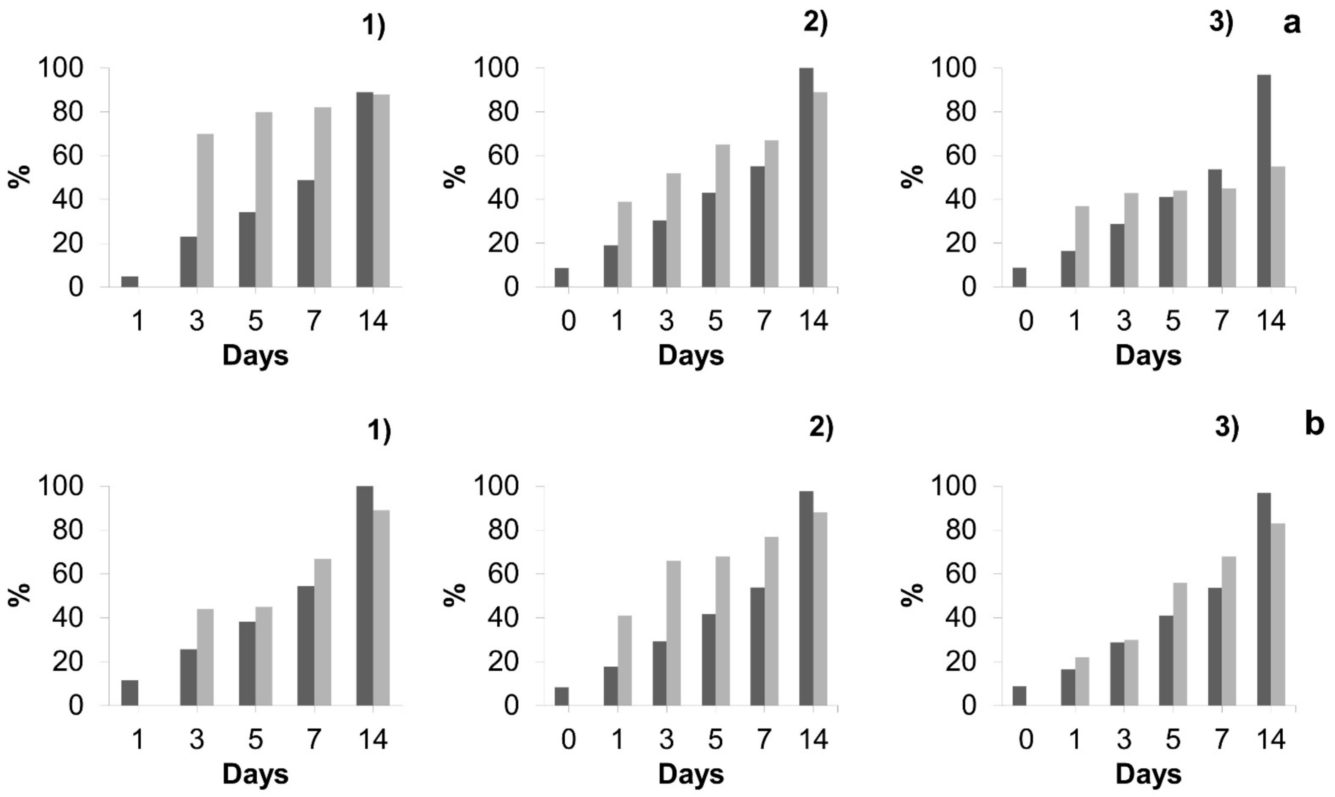


**Figure S4.** Cumulative $ET_0$ and cumulative dissipation of acetamiprid in jalapeno pepper in the greenhouse and open field. a) Greenhouse application 1) first application, 2) second application, 3) third application. b) Application in open field 1) first application, 2) second application, 3) third application. The dark gray bar refers to the accumulated $ET_0$, and the light gray bar refers to the percentage of acetamiprid residue.

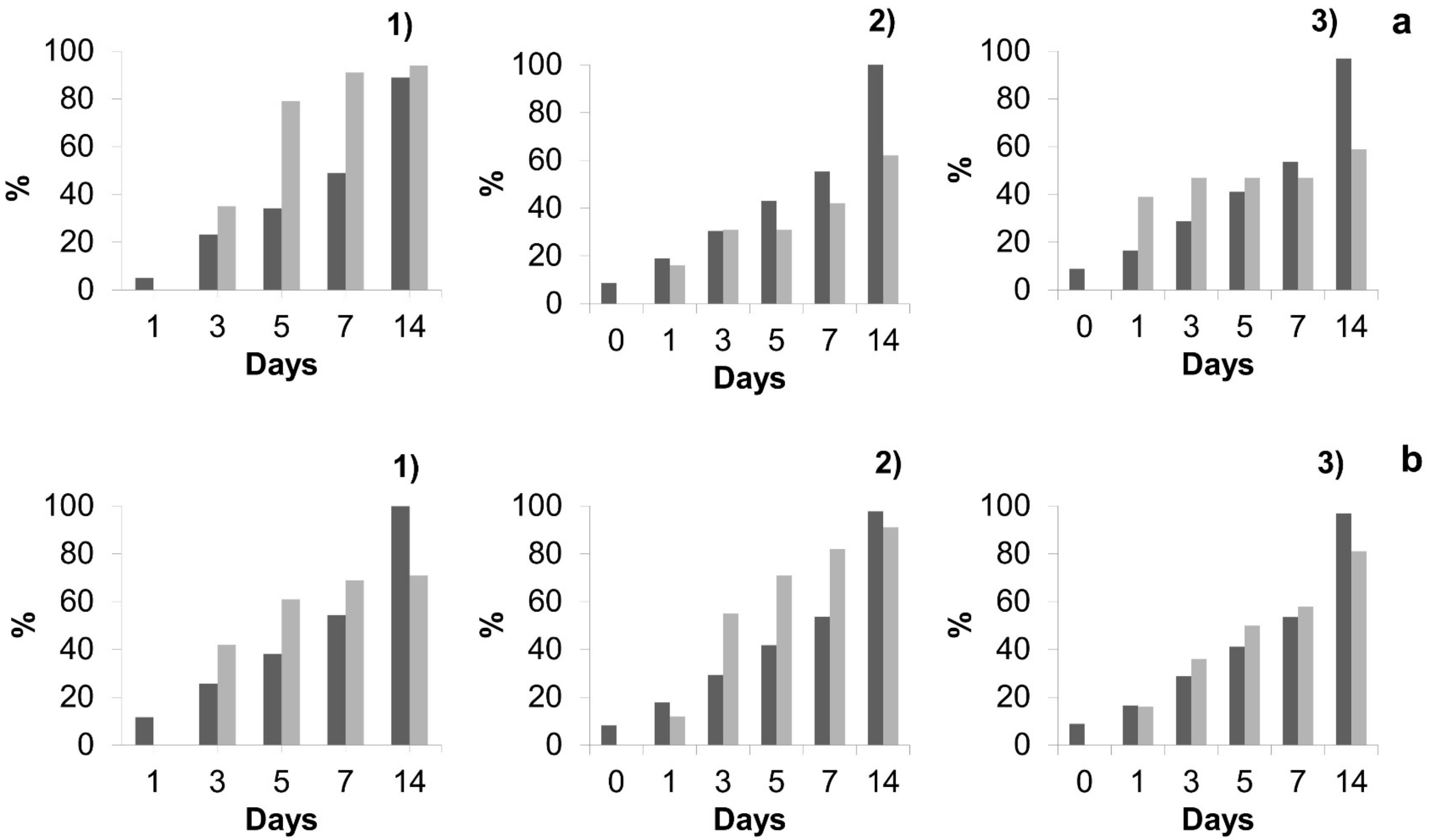


**Figure S5**. Cumulative $ET_0$ and cumulative dissipation of azoxystrobin in jalapeno pepper in greenhouse and open field. a) Greenhouse application 1) first application, 2) second application, 3) third application. b) Application in open field 1) first application, 2) second application, 3) third application. The dark gray bar refers to the accumulated $ET_0$, and the light gray bar refers to the percentage of azoxystrobin residue.

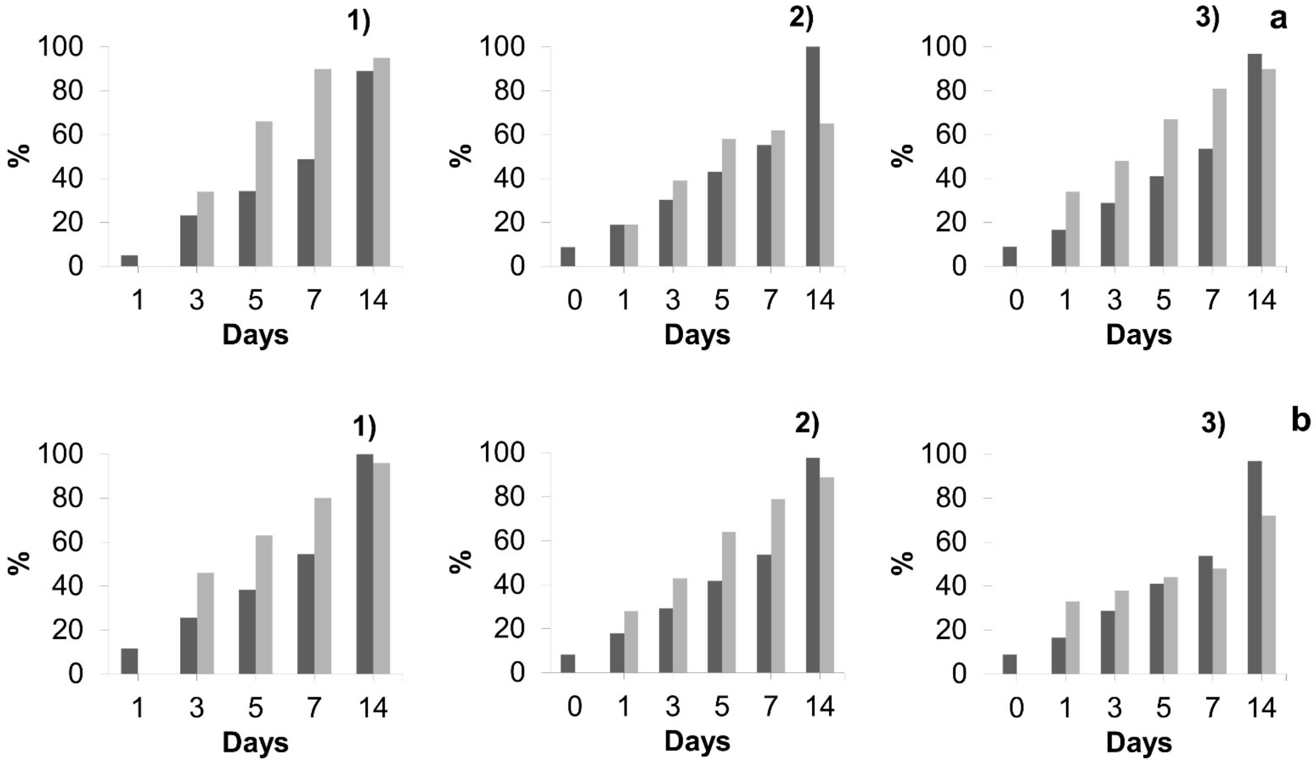


**Figure S6**. Cumulative $ET_0$ and cumulative dissipation of β-cyfluthrin in jalapeno pepper in greenhouse and open. a) Greenhouse application 1) first application, 2) second application, 3) third application. b) Application in open field 1) first application, 2) second application, 3) third application. The dark gray bar refers to the accumulated $ET_0$, and the light gray bar refers to the percentage of β-cyfluthrin residue.
